# Families with young children during the COVID-19 pandemic—The importance of family type, perceived partnership roles, parental stress, and social support for changes in the home learning environment during lockdown

**DOI:** 10.3389/fpsyg.2023.1119950

**Published:** 2023-02-07

**Authors:** Luisa Prokupek, Franziska Cohen, Elisa Oppermann, Yvonne Anders

**Affiliations:** ^1^Chair of Early Childhood Education, Institute for Educational Science University, University of Bamberg, Bamberg, Germany; ^2^Department of Early Childhood Education, Institute for Educational Science, University of Education Freiburg, Freiburg, Germany

**Keywords:** COVID-19, home learning environment, family adaptability, family type, perceived partnership roles, parental stress, social support

## Abstract

Beginning in March 2020, the lockdown precipitated by the COVID-19 pandemic resulted in many challenges, especially for families with young children. Many children had little or no access to institutional education. Therefore, they were even more dependent on their parents providing them with home learning activities (HLA) to support their development. We examined the adaptability of families with regard to changes in parents’ provision of HLA in traditional two-parent families, single parent families, and large families compared to before the lockdown. We focused on family resources, such as a supportive distribution of roles within the partnership, or social support, as predicting factors of adaptability in *N* = 8,513 families with children aged 18–69 months. In addition, we considered parental stress as a further influencing factor. The cross-sectional data depicts families from a nationwide online survey, which we conducted during spring 2020 in Germany. We found that (a) all three family types offered their children more learning activities at home, albeit with slight differences between the families. However, (b) we identified differences in the factors influencing families’ adaptability: Across all family types, we found slight to medium negative relations between adaptability and parental stress. The relations were most evident in large families. Furthermore, social support exhibits somewhat positive relations to the adaptability of large families. For adaptability in single-parent families, gender differences were initially evident. Among single fathers, the change in parental HLA was stronger than among single mothers. However, this relation disappeared when we took parental stress and social support into account. For traditional two-parent families and single parents, our analyses revealed (c) barely significant relations between the investigated predictors and changes in HLA during lockdown. Overall, our study confirms that high stress limits the adaptability of providing HLA in families and that social support mitigates negative relations between stress and the provision of HLA, especially in large families. In order to develop effective and needs-based family support programs, it is therefore important to help parents cope with stress and provide them with low-threshold social support. The extent to which these services need to be adapted to different family types must be surveyed in more depth.

## 1. Introduction

In the spring of 2020, the COVID-19 pandemic reached Germany, causing severe disruptions for families and their everyday lives. Parents with children under the age of six were particularly exposed to challenges compared to other parents and reported a substantial reduction in their satisfaction with family life (Huebener et al., 2020). One reason for this was the nationwide closure of daycare centers. As a result, many families were denied centralized care and education services. Moreover, contact restrictions also made other care options, such as grandparents, impossible. COVID-19 policies also influenced the working lives of parents (Cohen et al., 2020). Parents who were worried about diminished employment income due to, for example, reduced hours at work or even the loss of their job, had to deal with further challenges and financial concerns. Even those parents who were able to work from home still had to compensate for the missing hours of childcare within the family. Hence, parents had to deal with the difficulties posed by lack of childcare and the new demands of reconciling family and work. Families needed to adapt in the shortest possible time, reorganize working and childcare hours, and redistribute childcare at home. International research on the severe disruptions for families during the COVID-19 pandemic has increased considerably in just a few years. Still few studies focus specifically on families with young children (Huebener et al., 2020; Linnavalli and Kalland, 2021). Some international studies about families with young children examine the impact of the COVID-19 policies on parental and child well-being. The studies often focus on family strains, changes in parenting practices, child development (e.g., socio-emotional development) and family resilience (Gassman-Pines et al., 2020; Prime et al., 2020; Romero et al., 2020). In Germany, studies indicate that children and families may have been particularly vulnerable during the COVID-19. This findings show that family strains and coping to parental stress posed major challenges to parenting during the COVID-19 pandemic (Huebener et al., 2020; Essler et al., 2021; Oppermann et al., 2021; Calvano et al., 2022).

In view of the increasing complexity of the living environment and new societal demands on families, such as those resulting from the COVID-19 pandemic, it is becoming increasingly important to understand those factors that can influence the adaptability of families. In the present study, we consider family adaptability in terms of parents’ ability to ensure that children continue to receive sufficient educationally stimulating activities, even if the educational remit of early childhood education institutions cannot be carried out and other stressors impair the functionality of the family system. Another aim of the study is also to investigate family adaptability among traditional two-parent families, single parents, and large families during the first COVID-19 lockdown. This enables statements about adaptability to be linked to the ratio of children to adults within a family setting. In addition to the family type, we also take the relevance of a supportive role distribution within the partnership, social support, and parental stress into account. This family type characteristic approach should help us to learn more about the impact of COVID-19 restrictions on families with young children. Furthermore, we intend to identify protective and risk factors and formulate family type-specific conditions for adaptability.

## 2. Adaptability of the home learning environment

**Figure 1:**
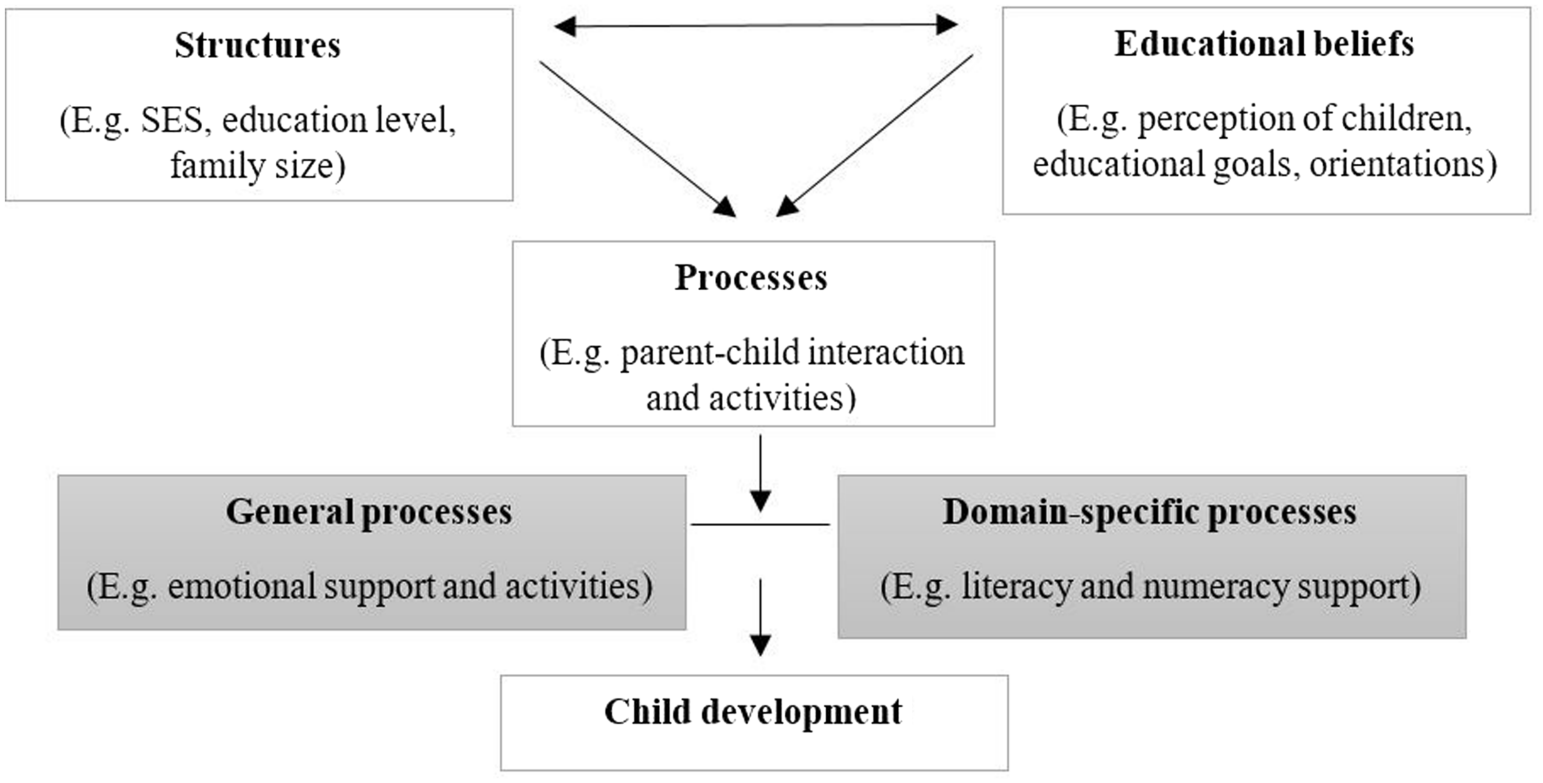
Framework of the HLE (Kluczniok et al., 2013).

Referring to Bronfenbrenner (1986) eco-systemic model of human development, the family is a learning environment with its own specific characteristics. In order to describe the processes within a family, and the effects they have on child development, the framework of the home learning environment (HLE), shown in Figure 1, summarizes structural, processual, and orientation dimensions (Kluczniok et al., 2013). These three dimensions – partly indirectly and partly directly – influence child development, and represent a way of categorizing resources in the learning environment of a family. The structural dimension summarizes characteristics such as household income or parents’ educational level, and interrelates with characteristics of the orientation dimension, which describes parents’ attitudes to education and child-rearing. The family type (e.g., single parent, two-parent family, large family), which is made up of different personal resources (number of adults and children within a household), is also understood as a characteristic of the structural dimension. Both structural and orientation characteristics are related to the availability of educational resources, such as parent–child activities. We describe these as home learning activities (HLA). The processual dimension includes global family processes such as day-to-day activities or the fostering of a familial atmosphere, but also domain-specific family processes, which focus on educationally relevant activities (Kluczniok et al., 2013). Process quality is determined *via* the frequency and quality of stimulating activities in the home, specifically educationally relevant activities (e.g., reading aloud or looking at picture books together). Studies show a clear, but moderate, relation between structural characteristics and family process quality. Thus, lower socio-economic background characteristics (e.g., low levels of parental education) in a family are associated with a lower quality of processes in families (Sylva et al., 2004; Melhuish et al., 2008).

**Figure 2:**
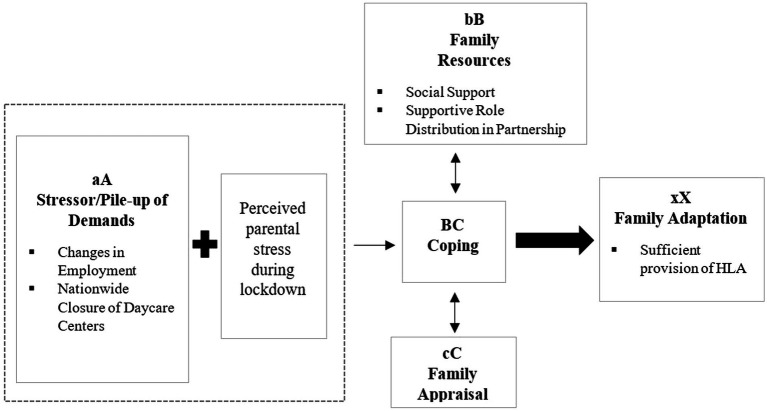
Own depiction according to the double ABC-X-Model of family adaptation (McCubbin and Patterson, 1983).

Family life is subject to certain dynamics. Interactions and relationships between family members respond to changing internal and external circumstances (Bradshaw et al., 2006). If the living conditions of families change (e.g., due to challenging life situations), this can have an impact on the HLE. For many years, family research looked at the special dynamics of families in challenging life situations with the help of the model of family adaptation (McCubbin and Patterson, 1983), shown in Figure 2. From the perspective of child development, appropriate family adaptability must consist of ensuring the educationally supportive character of the HLE, even in challenging life situations or when it is not possible for the child to attend the relevant educational institutions. Thus, in order to understand the needs and adaptability of families, it seems reasonable to assume a link between the HLE and factors which influence family adaptability. The model of family adaptation presents four components of families’ adaptability (A, B, C, and X) to a critical event and addresses the interaction between family resources and family perceptions. From this point of view, we can conclude that it is not only the stressor (critical event) that leads to a family crisis. Rather, it depends on whether and in what way a family is able to react appropriately to the stressor and adapt to the concomitant changed life circumstances: The family might equally develop adequate adaptation strategies to deal with the stressful life event and thereby maintain functionality as a family unit (Xu, 2007). The process of family adaptation to stressful life events consists of an interplay between the four different components A, B, C, and X. Stressor A, e.g., the impact of the interventions taken during the COVID-19 pandemic**—**such as changes in employment or the nationwide closure of daycare centers**—**disturbs the family’s previous patterns of interaction. The internally and externally available resources (e.g., supportive role distribution in the partnership or social support; B) enable families can respond to the stressor. The appraisal of the stressor (e.g., threat or challenge; C) also influences the coping strategies a family uses to respond to the stressor (BC). The outcome of the interactions between these identified variables corresponds to the family’s adaptability (here, the appropriate provision of HLA, xX). The model focuses on the evolving nature of the family unit, including general stressors of everyday family life. It takes into account additional stressors that may exacerbate the stress that families already face in their daily live (aA). The same applies to expanded resources (bB) and changes in the meaning attributed to a challenging situation (cC) (McCubbin and Patterson, 1983). The Double ABC-X Model of family adaptation shows conceptual similarities to the Family Stress Model (Masarik and Conger, 2017). Both models focus on similar aspects, but differ primarily in the arrangement of variables. The Family Stress Model focuses on stress processes of families, whereas the Double ABC-X Model describes the process of everyday adaptation as well as adaptation during times of crisis. We include the influence of stress on parenting behavior in the Double ABC-X Model and consider this aspect as a variable influencing family adaptability.

During the first lockdown, children had little or no access to institutional education for several months. During this time, they were more dependent than usual on their parents’ provision of HLA to stimulate their development. We therefore consider successful adaptability in families during the first lockdown of the COVID-19 pandemic as being represented by parents provided sufficient HLA for their children, even when the attendance of the child’s educational institutions was not possible and the functionality of the family system is impaired by other stresses.

### 2.1. Family adaptability: Protection and risk factors

The challenges of everyday life, as well as changing demands within society, require families to adapt permanently. The concept of family adaptability is also applied when it comes to specifying the change processes which take place in families in response to challenging life situations or crises (Moen and Wethington, 1992) and therefore also for the challenges of family life during the COVID-19 pandemic. Fundamental to understanding family adaptability is the identification of protective factors such as internal family resources (e.g., supportive role distribution within the partnership), financial resources, and social support (Patterson, 2002). These factors all affect a family’s involvement in children’s development, as well as influencing their broader educational decisions (Kluczniok et al., 2013).

Parental stress experiences, as well as adaptation strategies, influence how intensively parents offer their children HLA (Wierda-Boer et al., 2008; Holthus and Bertram, 2018). It is not only the frequency of educationally stimulating parent–child interactions that matters, but also the parents’ responsive and sensitive interaction behavior (Anders et al., 2012; Kluczniok et al., 2013). More and more studies investigating the factors influencing parenthood include measures of parents’ perceived stress levels (Crnic and Ross, 2017). According to Deater-Deckard (2004), parental stress describes processes that lead to aversive reactions. The origin of these reactions is the attempt to adapt to the demands of parenthood. The perception of parental stress and the effects of it reflect systemic processes within the family (Crnic and Ross, 2017). Many studies suggest that parental stress predicts inadequate parenting behavior (Crnic and Greenberg, 1990; Crnic et al., 2005). For example, research on the influence of economic stress shows that parents are irritable, frustrated, and less patient when emotional resources cannot be accessed during times of financial stress. The consequences are more punitive parenting as well as the withdrawal and social alienation of the stressed parent (McLoyd, 1990; Magnuson and Duncan, 2002). An earlier study by Oppermann et al. (2021) on the same sample as this article investigated the correlation between parental stress and changes in HLA during the first COVID-19 lockdown in Germany. They found a negative, exponential relation, indicating a threshold of parental stress: The relation between perceived parental stress and changes in HLA appeared above a certain tipping point. Past this parental stress level, HLA exponentially decreased with increasing stress.

The identification of family resources (e.g., supportive role distribution within the partnership) plays an important role in adaptability (Patterson, 2002). In times of crisis, responsibilities for employment, household, and child-rearing have to be (re-)distributed. This can have a great influence on parental well-being (Fankhauser et al., 2018). Consequently, the supportive role distribution within the partnership as a predictor of parental well-being plays an important role in the functioning of families and the development of children in their home environment. We therefore want to investigate to what extent the supportive role distribution functions as an intra-family protective factor for the adaptability of families (maintaining or increasing HLA provision) in times of crisis. It is just as important that parents feel supported by their social environment and know where to ask for help. Studies have identified social support outside the family as an underlying mechanism for successful adaptability. It contributes significantly to how strongly a family feels the emotional burden of a crisis (Dunn et al., 2001; Wierda-Boer et al., 2008; Manning et al., 2011). Most recently, Oppermann et al. (2021) showed in their family survey that, during the first lockdown, social support was associated with lower parental stress. Furthermore, social support reduced the negative effects of stress on HLA, highlighting the importance of social support for family adaptability.

### 2.2. Why family types matter

A look at family research shows that most studies deal with the family as a generic unit and rarely classify different family types. Studies often focus on heterosexual married or cohabiting couples and their children (two-parent families; Wierda-Boer et al., 2008; Manning et al., 2011; McStay et al., 2014; Liu et al., 2019). Childhood and family also take place in non-heterosexual relationships. In addition, families form through surrogacy, permanent foster care, or adoption. Moreover, family structures can change over the course of life, regardless of how they are founded (Riggs and Due, 2018). Studies that look at the importance of different family types for child development increasingly examine whether certain family types are more affected by disadvantages than others. According to Bradshaw et al. (2006), children from large families are affected by a greater risk of poverty than children from other family types – poverty can expose families to financial crisis. It is known from current research that in large families, for example, due to the high number of children, that the likelihood of only one parent being employed is higher, while the other parent is more likely to be responsible for care and nursing activities (Bradshaw et al., 2006). Research on single parents, on the other hand, shows that single parents are more likely to have lower parental SES, because SES often reduces after separation or divorce due to the lack of a second income (OECD, 2018). When it comes to coping with everyday demands, studies showed that single parents in particular face special challenges, due to their single-parent status and the reduced time and social resources in their family structure that often go hand in hand with this (Wright, 1989; Bowen et al., 1993). Some studies also argue that if single parents have few opportunities for social support, then these families are more vulnerable to family stress (McQuillan and Bates, 2017). As early as the 1990s, Bowen et al. (1993) studied the adaptability of single parents and found that the lack of social support was the greatest risk factor among single parents in this context. Studies that are more recent also follow these findings (Maldonado and Nieuwenhuis, 2015; Jackson and Kiehl, 2017; Martin-West, 2019). Educational research has not yet sufficiently mapped out the extent to which the factors influencing adaptability differ in other family types. This raises the question of whether it is not precisely families with special needs that face particular challenges in times of crisis, or whether, based on the family type, different resources must be drawn on to cope with challenging life situations. However, in view of the increasing complexity of the living environment and new societal demands on families, it is becoming more and more important to understand those factors that can influence the adaptability of families.

### 2.3. Purpose of the study and research questions

In Germany, the COVID-19 lockdown confronted families with challenging situations. Families with young children in particular faced major challenges during this time because children in this age group need more intense care. In addition, they are highly dependent on stimulating HLA offered by their parents, as there is still no influence from the school system. The closure of daycare centers in particular resulted in new demands on the reconciliation of family and work. Some parents also had to cope with changes in their employment (Cohen et al., 2020; Huebener et al., 2020). Those parents who were less affected by changes in employment still had to make up for the missing hours of childcare outside the home. The challenge of continuing to cope with everyday family life also was noticeable in parents’ perception of stress (Oppermann et al., 2021).

The number of studies investigating the challenges of family life during the COVID-19 pandemic has increased significantly in recent times. However, since many of the studies deal with the family as a general unit, we would like to focus on different types of families. The negative relation between parental stress and the HLE in general and especially during the first COVID-19 lockdown has already been scientifically proven (Crnic and Greenberg, 1990; Crnic et al., 2005; Oppermann et al., 2021). However, we also want to investigate whether these findings apply equally to all family types. In addition, we are interested in whether negative relations between parental stress and changes in HLA occur in the different family types from the same stress level (low, medium, high levels of stress) onwards. In order to identify, among the surveyed families, those types with special needs in times of crisis, we investigate whether social support is equally considered a protective factor for family adaptation in all families. Finally, we want to look at supportive role distribution in couple households under a family type-specific focus and explore whether the importance of a supportive role distribution within the partnership for adaptability (Patterson, 2002) differs between traditional two-parent families and large families.

Based on current data, we examine differential links between family adaptive capacities (changes in HLA); taking into account different family types (two-parent families, large families, and single parents). Due to different family types, our aim is to examine the relations of changes in parents’ HLA with their children during the first COVID-19 lockdown. We define positive adaptability as maintaining or increasing the parental level of HLA with preschool children. This approach aims to better capture the protective factors of families, in addition to the impact of the COVID-19 lockdown, on families with young children, and thus formulate family type-specific needs in crises. We define the following research questions (RQ):

1. What is the impact of the first COVID-19 lockdown on adaptability of parents’ HLA in different family types?
  - We expect families to draw on different resources based on their family type and therefore we will find differences in adaptability of parents’ HLA (H1a).
  - We assume that the change in employment as well as in external childcare is related to the adaptability of families (H1b).
2. What is the importance of perceived roles in the partnership, parental stress, and social support for changes in the home learning environment?
  - We assume positive relations between (1) the supportive distribution of roles within the partnership and (2) social support and family adaptability (H2a1 and H2a2).
  - We assume that parental stress influences the adaptability differently in the family types (H2b).
  - We assume that the relations of perceived stress and adaptability are strongest in single-parent families because of the lower personal resources (only one adult within a household) (H2c).
  - We assume that the relations of perceived stress and adaptability will be lowest in large families due to the distribution of employment and care work for mostly several young children (H2d).

## 3. Methods

### 3.1. Design and sample

Data stems from the study ‘Families and Childcare centers in times of COVID-19’, a nation-wide cross-sectional online survey, which examined the effects of the abrupt closure of daycare centers during the first COVID-19 lockdown on German families with young children (Cohen et al., 2020). However, since the restrictions differed greatly in Germany’s various federal states, we cannot assume that the sample is representative of the whole of Germany. To our knowledge, this was one of the first studies to examine the situation of families with young children during the first wave of the COVID-19 pandemic in Germany. In April and May 2020, 9,343 parents whose children attended daycare before the lockdown participated in the survey. We recruited a convenience sample through personal contacts, online blogs, social media, and the mailing lists of large non-profit organizations, foundations, and daycare centers. The recruited parents came from all 16 German states, with clustering in some federal states like Bavaria, Baden-Württemberg and North Rhine-Westphalia (Cohen et al., 2020). We excluded all cases without children in the household (*n* = 289). We did the same with those who had a relationship with the child other than mother/stepmother or father/stepfather (*n* = 181). Finally, we excluded single-parent families who lived with other family members or with roommates/friends from the sample (*n* = 360). Thus, the sample does not represent all family types living in Germany. To answer the research question *N* = 8,513 families with children aged 18–69 months (*M* = 37.0, *SD* = 4.5) from the main sample fit these criteria. Of these, 89% of respondents were mothers (*n* = 7,551). The average age of respondents was 37 years (*SD* = 4.5; age range 18–69 years). We determined the parental educational level *via* three levels: *low*, which corresponds to ISCED levels 0–2 (lower secondary school education and below), *medium*, which corresponds to ISCED levels 3–5 (upper secondary school education to short-cycle tertiary education) and *high*, which corresponds to ISCED levels 6 and 7 (Bachelor’s degree or above; UNESCO, 2011). With regard to the level of education, we classify the families as privileged: In all three family types, the ISCED is close to the medium range (Single parents: *M* = 2.7, *SD* = 0.6; traditional two-parent families: *M* = 2.9, *SD* = 0.4, large families: *M* = 2.8, *SD* = 0.5). Moreover, 85% of parents in the sample had a high educational level, 13% had a medium educational level and 0.9% had a low educational level. At the time of the survey, 74% of parents were employed (21% fulltime, 45% parttime, and 8% on reduced hours). Before the COVID-19 pandemic, 79% of parents were employed (27% fulltime, 52% parttime, and 0.3% on reduced hours). The proportion of those who were on leave or looking for work also increased from 1% to 5% compared to before the lockdown and the proportion of those who regularly work from home increased from 28% to 38% compared to the situation before COVID-19. The employment situation of the respondents’ partners has also changed significantly, with from 96% in employment (82% fulltime, 12% parttime and 0.3% on reduced hours) before the lockdown, and 93% in employment during (68% fulltime, 13% parttime and 11% on reduced hours). In addition, 30% of respondents and 22% of partners worked in systemically relevant occupations, which gave them special entitlement to daycare during the lockdown. However, it should be emphasized that the classification of system-relevant occupations was quite heterogeneous between the federal states and at the time of the first daycare closure. At this point, the average time children spent in care outside the home dropped from 7 h/day (*SD* = 2.4) to 1.5 h/day (*SD* = 3.0). The surveyed families also hardly felt any stress due to the employment situation (their own or their partner’s; *M* = 3.0, *SD* = 1.3) or partnership conflicts (*M* = 2.6, *SD* = 1.1). Descriptive analyses of family cohabitation revealed that 93% of the families surveyed live together as a traditional two-parent family (*n* = 7,911). Five percent of the families are single parents (*n* = 429). In contrast, 2% of respondents live together in a large family (*n* = 173). Regarding parental education, all three family types show values on the medium ISCED level. The highest parental educational background was found in two-parent families (*M* = 2.9, *SD* = 0.4). Large families were close behind (*M* = 2.8, *SD* = 0.5). Among single parents, we found the lowest levels parental educational, albeit with only a slight difference (*M* = 2.7, *SD* = 0.6). On average, the families largely describe their financial situation before COVID-19 as unproblematic.

### 3.2. Measures

#### 3.2.1. Family type

In order to identify different family types, we established family type criteria. Due to the data situation, we are not able to depict all family types living in Germany. We defined the following family types: The “two-parent family” describes cases in which two parents lived together as a couple with one to three children as a family unit in a common household. “Large families” are cases in which two parents lived together as a couple with four or more children as a family unit in a common household (Toth et al., 2019). “Single parents” were defined in the study *via* cases where the respondent stated that he or she lives alone with his or her child(ren) in a common household. For our approach, the number of children living in the single-parent household was not relevant. We statistically excluded a crossing of the categories.

#### 3.2.2. Family adaptability

In order to be able to make statements about the current adaptability in families, we generated a change variable of the HLA-quantity compared to the time before the lockdown, which formed the dependent variable within our model specification. Using a 7-point response scale (1 = considerably less frequent; 4 = the same; 7 = considerably more frequent) we asked parents about the frequencies of educational activities (e.g., reading aloud or handicrafts) that they or another family member engaged in with the target child, compared to the time before the lockdown (*α* = 0.85). The focus of the survey was kindergarten children. Thus, the parents indicated how many children between 0 and 6 years of age live in their household. If there were several children belonging to this age group in a family, the parents were asked to self-report reading aloud or doing handicrafts with the oldest child within this age group.

#### 3.2.3. Predictors of changes in HLA

We measure *changes in employment at the household level* (−1 = less, 0 = no change, 1 = more) and *changes in hours of childcare outside the home* (−2 = significantly less, −1 = less, 0 = no change, 1 = more 2 = significantly more) compared to the time before the lockdown as reported by the families as predictors. Parental stress comprised four items, which indicated the level of perceived stress (e.g., “I often feel unable to cope with the new tasks and demands, e.g., home schooling, keeping the child occupied”) by using the scale mean from 1 to 4 with higher values indicating a higher parental stress level, (*α* = 0.85). To investigate the relation between parental stress and adaptability in detail, we followed the threshold concept of Oppermann et al. (2021) and additionally measured parental stress as dummy variables to include these in multiple group regressions: With the help of a quartile split, medium-stress and high-stress limits were determined. Parents between a value of 2.33 (2 = tend to disagree) and 3.0 (3 = tend to agree) on the linear stress scale are labelled with 1 in the dummy variable *medium stress* (1 = medium stress, 0 = low or high stress). Parents whose stress perception on the linear stress scale is at least 3.25 are labelled with 1 in the dummy variable *high stress* (1 = high stress, 0 = medium or low stress). We define *low stress* from a scale value of 2.25 or lower and use this variable as a reference group in later analyses. Four items assessed *social support*, which described how strongly the respondent felt supported by his or her social environment during the lockdown (e.g., “Is there someone who can give you good advice when you have problems?”). On a 5-point response scale, respondents could indicate the perceived availability of social support (1 = never, 5 = always). The reliability test resulted in a Cronbach’s alpha of.89. *Supportive role distribution within the partnership* consisted of four items, e.g., if the respondent felt sufficiently supported by his or her partner in childcare or in the household. A 4-point response scale was used to measure agreement with these statements (1 = strongly disagree, 4 = strongly agree). Single parents could not answer this scale because of their single status. The scale proved to be reliable (*α* = 0.86).

#### 3.2.4. Control variables

As control variables, we implement parental education (ISCED level: *low* = 0–2, *medium* = 3–5, *high* = 6–7), the perceived financial problems before COVID-19 (1 = we had no financial problems, 5 = we had great financial worries), the gender of the respondent (0 = female, 1 = male), as well as the child’s age (in months) at the time of the interview and the gender of the target child (0 = female, 1 = male).

### 3.3. Statistical analyses

To investigate the impact of the first COVID-19 lockdown on the adaptability of parental HLA in different family types (RQ1), we compute descriptive statistics and use analyses of variance with *post hoc* analyses. Thus, we test the extent to which the mean values of parents’ HLA, the change in employment as well as in external childcare differ significantly among the three family types. To test, whether the changes in employment and in external childcare are related to the adaptability of families, we conduct multiple group regressions in Mplus, with the family type being the grouping variable (Version 8.3; Muthén and Muthén, 1998–2012). Multiple group analysis allows us to test if pre-defined data groups (family type) have significant differences in their group-specific parameter estimates (e.g., standardized regression coefficients, standard errors and significance for family resources, perception of parental stress and social support). To answer the research question regarding the importance of perceived roles in the partnership, parental stress, and social support for changes in the home learning environment (RQ2), we initially compute descriptive statistics and analyses of variance. In addition, we investigate relations between the supportive distribution of roles within the partnership and changes in parents’ HLA using further multiple group regressions combined with post hoc analysis of the regression coefficients (Wald-Test). We survey relations between social support and changes in parental HLA in a similar way. To test the importance of parental stress for changes in the home learning environment we additionally use further multiple group regressions combined with post hoc analysis of the regression coefficients.

To verify whether the data of the relevant variables were systematically missing, we conducted a missing data analysis in all cases. We used Little’s (1988) test for completely missing values at random (MCAR). The results of the MCAR test showed that data were not missing at random (MNAR) in the continuous variables relevant to us (e.g., perceived financial problems, changes in employment, or in hours of childcare outside the home: *χ*^2^ = 141.71, *df* = 54, *p* = 0.000). However, Shin et al. (2009) argue that even under MNAR, the full information maximum likelihood (FIML) approach yields the least biased parameter estimates and the lowest parameter estimation error. To make full use of the data, we used FIML in all our analyses to answer our research question. The model fit was assessed with reference to the Yuan-Bentler scaled *χ*^2^ (YB *χ*^2^, mean-adjusted test-statistic robust to non-normality), the root mean square of approximation (RMSEA), the comparative fit index (CFI), the Tucker and Lewis index (TLI), and the standardized root mean residual (SRMR) values according to the criteria recommended by Hu and Bentler (1999). CFI and TLI values >0.95, RMSEA values lower than 0.06, and SRMR lower than 0.08 were accepted as indicators of a good model fit (Hu and Bentler, 1999).

## 4. Results

### 4.1. Descriptive findings

In all three family types, there were hardly any changes in employment during the first lockdown. This does not mean that families were not burdened by changes. Even though the amount of employment did not change noticeably, the percentage of respondents working from home increased from 28% to 38% during the first lockdown. In all families, there was a noticeable decrease in hours of care outside the home during the lockdown. Single parents (*M* = −1.4, *SD* = 0.9) were slightly less affected by these changes than two-parent families (*M* = −1.7, *SD* = 0.7). We can explain this in part by the fact that single parents had a special entitlement to emergency daycare. Also, large families (*M* = −1.5, *SD* = 0.9) were slightly less affected by these changes than two-parent families (*M* = −1.7, *SD* = 0.7). All families, regardless of family type, were equally affected by parental stress. We observe the same for the perception of social support: All families reported an intermediate level of social support. Two-parent families and large families alike reported living in a partnership with a tendency toward a supportive distribution of roles. In all three family types, parents provided HLA somewhat more frequently on average, which corresponds to the value 5 on the response scale, during the first lockdown (see Table 1). Here, however, in comparison, large families offered the least (*M* = 4.6, *SD* = 1.0), less than single parents (*M* = 4.9, *SD* = 0.8) and two-parent families (*M* = 5.0, *SD* = 0.8).

**Table 1:** Descriptive statistics for family types.

	Single parents (*n* = 429)		Two-parent families (*n* = 7,911)		Large families (*n* = 173)	
	*M*	*SD*	*M*	*SD*	*M*	*SD*
Age of child (in months)	37.0	5.4	37.0	4.5	39.0	4.2
Perceived financial problems before COVID-19 (1 = no financial problems; 5 = major financial problems)	2.0	1.0	1.4	0.7	1.7	0.9
ISCED	2.7	0.6	2.9	0.4	2.8	0.5
Change in employment (household level; 1 = more, 0 = unchanged, −1 = less)	−0.2	0.4	−0.3	0.5	−0.3	0.5
Change in hours of childcare outside the home (2 = considerably more, 0 = unchanged, −2 = considerably less)	−1.4	0.9	−1.7	0.7	−1.5	0.9
Parental stress (1 = totally disagree, 4 = totally agree)	2.7	0.8	2.7	0.7	2.7	0.7
Low stress (0 = medium or high stress, 1 = low stress)	0.3	0.5	0.3	0.5	0.3	0.5
Medium Stress (0 = low or high stress, 1 = medium stress)	0.4	0.5	0.4	0.5	0.4	0.5
High stress (0 = medium or low stress, 1 = high stress)	0.3	0.5	0.3	0.5	0.3	0.5
Social support (1 = never, 3 = sometimes, 5 = always)	3.3	1.0	3.3	1.0	3.2	1.1
Supportive role distribution partnership (1 = totally disagree at all, 4 = totally agree)	–	–	3.1	0.7	3.1	0.8
Changes in parents’ HLA with their children (1 = considerably less frequent, 4 = Same, 7 = considerably more frequent)	4.9	0.8	5.0	0.8	4.6	1.0

### 4.2. Predicting changes in parents’ HLA in different family types

First, we consider the relations of the interventions taken during the COVID-19 pandemic (aA component: pile-up of demands): The changes in employment and in hours of childcare outside the home are additional stressors that add to the stress that families already face in their daily lives. Taking into account control variables, we examine the relations changes in employment and in hours of childcare outside the home exhibit with HLA (Model 1). The interventions taken during the COVID-19 pandemic may increase parents’ perception of stress (aA component: pile-up of demands). Model 2 tests relations between parental stress and HLA, focusing on particularly stressed families. To understand more about the relevance of available external resources (bB), we analyze relations between social support and HLA in Model 3. This allows us to explore the extent to which the availability of social support mitigates stress relations. In order to gain more knowledge about the importance of available internal resources (bB), we examine the relation between supportive role distribution and HLA in Model 4. The question as to the extent to which supportive role distribution functions as a family-internal protective factor during times of crisis can thus be investigated. Using this model specification, we explain relations between the relevant variables and the adaptability of families (maintaining or increasing HLA provision) in times of crisis. We must point out that we do not have assessable data for the component cC “familial appraisal.”

#### 4.2.1. RQ1: The impact of the first COVID-19 lockdown on adaptability of parents’ HLA in different family types

To analyze differences in adaptability of parents’ HLA based on the family type (H1a), we computed analyses of variance, which revealed differences in changes in parents’ HLA with their children (*F* (2, 7,549) = 12.93, *p* < 0.010, *η^2^* = 0.00,). Changes were significantly higher in two-parent families than in large families (*p* = 0.000). During the lockdown, parents from two-parent families offered HLA significantly more often to their children than parents from large families. Also, single-parent families offered HLA significantly more often than parents from large families (*p* = 0.010). We found no significant differences between two-parent families and single parents (*p* = 0.335). This confirms the established assumption.

To test hypothesis H1b, we investigated potential relations between changes in employment as well as in hours of childcare outside the home and the adaptability of families. Taking the control variables into account, the analysis of variance revealed significant differences between the groups with regard to changes in employment (*F* (2, 8,443) = 8.57, *p* < 0.010, *η^2^* = 0.00). In two-parent families, changes (reduction in hours worked) were significantly greater than in single-parent families (*p* = 0.000). In large families, changes (reduction in hours worked) were significantly greater than in single-parent families (*p* = 0.000), too. We found no differences between two-parent families and large families (*p* = 0.734). The ANOVA further shows differences in changes in hours of childcare outside the home between the family types (*F* (2, 5,556) = 25.98, *p* < 0.010, *η^2^* = 0.01). In two-parent families changes were significantly greater than in single-parent families (*p* = 0.000). We found neither differences between two-parent families and large families (*p* = 0.192) nor between single parents and large families (*p* = 0.322). Table 2 shows the results of the multiple group regression analyses. Including the control variables, changes in employment and in hours of childcare outside the home model 1 does not support our hypothesis H1b that changes in employment as well as in hours of childcare outside the home are related to the adaptability of families. In two-parent families, we found significant relations between all variables and changes in parents’ HLA. However, the relations are too small to interpret. Moreover, the large sample of 7,884 two-parent families is the reason for the significance of the findings. At 2%, the model explains little variance. In large families, we found no predictions of changes in parents’ HLA during lockdown by the control variables, changes in employment, or in hours of childcare outside the home. For large families, the model explains 5% of variance. For single parents, the model showed small relations between the gender of the parent and changes in HLA: Single fathers (*n* = 19) reported greater increases in HLA during the first lockdown than single mothers did. The model explains 5% of the variance. With the exception of the CFI/TLI values, the model fit was acceptable (*χ*^2^ = 203.32, *df* = 30, RMSEA = 0.05, CFI = 0.00, TLI = 0.00, SRMR = 0.03). This does not confirm the assumption made.

**Table 2:** Multi-group regression: changes in parents’ HLA in different family types.

	Model 1	Model 2	Model 3	Model 4
	*ß* (SE)	*ß* (SE)	*ß* (SE)	*ß* (SE)
Single parents (*n* = 428)				
Gender respondent (0 = female, 1 = male)	**0.11 (0.05)**	0.09 (0.05)	0.09 (0.05)	
Age child (in months)	−0.11 (0.06)	−0.09 (0.06)	−0.10 (0.06)	
Gender child (0 = female, 1 = male)	−0.09 (0.05)	−0.09 (0.05)	−0.09 (0.05)	
Perceived financial problems COVID-19	−0.04 (0.06)	−0.02 (0.06)	−0.02 (0.06)	
ISCED	0.07 (0.07)	0.06 (0.07)	0.06 (0.07)	
Change in employment at household level	−0.05 (0.05)	−0.04 (0.05)	−0.03 (0.05)	
Change in hours of childcare outside the home	−0.09 (0.08)	−0.10 (0.08)	−0.10 (0.08)	
Medium stress (0 = Low or high stress, 1 = Medium stress)		−0.04 (0.05)	0.04 (0.05)	
High stress (0 = Medium or low stress, 1 = High stress)		**−0.18 (0.06)**	**−0.19 (0.06)**	
Social support			−0.03 (0.06)	
*R*^2^	0.05	0.08	0.08	
Two-parent families (*n* = 7.884)				
Gender respondent (0 = female, 1 = male)	**0.03 (0.01)**	**0.03 (0.01)**	**0.03 (0.01)**	**0.03 (0.01)**
Age child (in months)	**−0.06 (0.01)**	**−0.06 (0.01)**	**−0.05 (0.01)**	**−0.05 (0.01)**
Gender child (0 = female, 1 = male)	**−0.04 (0.01)**	**−0.04 (0.01)**	**−0.04 (0.01)**	**−0.04 (0.01)**
Perceived financial problems COVID-19	**−0.06 (0.01)**	**−0.05 (0.01)**	**−0.05 (0.01)**	**−0.05 (0.01)**
ISCED	**0.03 (0.01)**	**0.04 (0.01)**	**0.04 (0.01)**	**0.04 (0.01)**
Change in employment at household level	**−0.04 (0.01)**	**−0.04 (0.01)**	**−0.04 (0.01)**	**−0.04 (0.01)**
Change in hours of childcare outside the home	**−0.07 (0.02)**	**−0.08 (0.02)**	**−0.09 (0.02)**	**−0.09 (0.02)**
Medium stress (0 = Low or high stress, 1 = Medium stress)		**−0.03 (0.01)**	−0.02 (0.01)	0.01 (0.01)
High stress (0 = Medium or low stress, 1 = High stress)		**−0.14 (0.01)**	**−0.11 (0.01)**	**−0.11 (0.01)**
Social support			**0.08 (0.01)**	**0.08 (0.01)**
Supportive role distribution				0.02 (0.01)
*R*^2^	0.02	0.04	0.04	0.04
Large families (*n* = 172)				
Gender respondent (0 = female, 1 = male)	0.03 (0.05)	0.04 (0.04)	0.04 (0.05)	0.03 (0.05)
Age child (in months)	0.04 (0.09)	0.04 (0.08)	0.02 (0.08)	0.02 (0.08)
Gender child (0 = female, 1 = male)	−0.03 (0.08)	−0.02 (0.08)	−0.01 (0.08)	−0.01 (0.08)
Perceived financial problems COVID-19	0.06 (0.09)	0.09 (0.09)	0.11 (0.09)	0.11 (0.09)
ISCED	−0.12 (0.09)	−0.16 (0.10)	−0.17 (0.09)	−0.17 (0.09)
Change in employment at household level	−0.10 (0.07)	−0.04 (0.07)	−0.04 (0.07)	−0.04 (0.07)
Change in hours of childcare outside the home	−0.13 (0.14)	−0.07 (0.13)	−0.13 (0.15)	−0.14 (0.14)
Medium stress (0 = Low or high stress, 1 = Medium stress)		−0.14 (0.08)	0.10 (0.08)	0.10 (0.08)
High stress (0 = Medium or low stress, 1 = High stress)		**−0.38 (0.07)**	**−0.27(0.09)**	**−0.27 (0.09)**
Social support			**0.25 (0.09)**	**0.25 (0.10)**
Supportive role distribution				0.01 (0.07)
*R*^2^	0.05	0.19	0.23	0.24

Displayed are standardized regression coefficients, standard errors in brackets, significance and explained variance. The bold values represent the significant results. The dataset contains 29 cases with missing on x-variables. These cases were not included in the analysis.

#### 4.2.2. RQ2: The importance of perceived roles in the partnership, parental stress, and social support for changes in the home learning environment

The surveyed families were slightly affected (2 = not at all stressful; 3 = somewhat stressful) by general stressors caused by the COVID-19 interventions, such as worries about children’s development (*M* = 2.8, *SD* = 1.3) or family health (*M* = 2.6, *SD* = 1.2). We used analysis of variance to examine differences in parental perception of stress between the family types: It revealed no significant differences between the families for linear parental stress (*p* = 0.727), low parental stress (*p* = 0.877), medium parental stress (*p* = 0.516), or high parental stress (*p* = 0.637). Then we included parental stress in our multiple group regression (see Table 2, model 2). In order to include particularly stressed parents among the families, we computed two dummy variables: one indicating intermediate levels of stress and one indicating high levels of stress. The analyses show no statistically significant relations for intermediate stress and changes in HLA among the families. Still, in large families there is a tendency toward a significant, but slightly negative relation between medium stress and changes in HLA (*p* = 0.082). Regarding high stress, the analysis reveals that the more stressed single parents were, the less they were able to offer their children the same level of HLA as before the lockdown. The gender differences among single parents disappear when we considered stress. The Mann–Whitney-*U* Test shows differences in the mean values for medium and high stress of single mothers and single fathers. Fathers (*M* = 0.6, *SD* = 0.5) are more affected by medium stress than mothers (*M* = 0.4, *SD* = 0.5). There was a tendentially statistically significant difference between the groups (*U* = 2,529, *Z* = −1.80, *p* < 0.10). Mothers (*M* = 0.3, *SD* = 0.5) are more affected by high stress than fathers (*M* = 0.1, *SD* = 0.2), There was a statistically significant difference in high stress between them (*U* = 2,468, *Z* = −2.11, *p* < 0.05). The model explains slightly more variance (8%) for this family type. For traditional two-parent families, due to the sample size, again almost all relations were statistically significant, but with very little to no relations. Only for relations between high parental stress and changes in HLA did the regression show that the more stressed traditional two-parent families were, the less the frequency of HLA changed. The model still explains little variance (4%). In large families compared to the other family types, the level of parental stress was of greater importance for an adequate adaptability of the HLA during the first lockdown. We found an intermediate negative relation between high stress and changes in parents’ HLA. This model explains significantly more variance (19%) than Model 1. The model fit was acceptable (*χ^2^* = 259.47, *df* = 27, RMSEA = 0.15, CFI = 0.00, TLI = 0.00, SRMR = 0.07). *Post hoc* analysis of the regression coefficients of high parental stress between the groups shows no differences between traditional two-parent families and single parents (*χ*^2^(1) = 0.865, *p* = 0.352, *d* = 0.021). We also find no differences between single parents and large families (*χ*^2^(1) = 2.929, *p* = 0.087, *d* = 0.038). Only between two-parent families and large families do the regression coefficients of high stress experience differ (*χ*^2^(1) = 6.247, *p* = 0.012, *d* = 0.056). In general, the results confirm hypothesis H2b, according to which parental stress influences the adaptability differently in the family types. However, hypothesis H2c assumed that the negative relations of perceived stress and adaptability are strongest in single-parent families because of their lower personal resources. Our results refute this. When we considering the regression coefficients, it becomes clear that negative relations between parental stress and changes in HLA are significantly greater in large families than in traditional two-parent families. Accordingly, we must reject the assumption that the negative relation between perceived stress and adaptability are lowest in large families (H2d).

To test hypothesis H2a2, we investigated relations between social support and family adaptability. In general, we found no statistically significant differences between the groups for social support (*p* = 0.479). When we included social support to the multiple group regression, we found no significant relations between social support and changes in parents’ HLA compared to the time before the lockdown for single parents (see Table 2, model 3). The variance reached its maximum at 8%. Although in traditional two-parent families the regression coefficient for social support is relatively small, the relations between high levels of stress and changes in HLA decrease when we consider social support. The variance remains at 4%. In large families, the availability of social support was more important for changes in HLA during the first lockdown compared to the other family types. For social support, the negative relations between high levels of stress and changes in HLA decrease (see Table 2, model 3). This model explains 23% of the variance. The model fit was acceptable (*χ^2^* = 296.30, *df* = 30, RMSEA = 0.14, CFI = 0.00, TLI = 0.00, SRMR = 0.07). Comparative post hoc analyses of the regression coefficients between the groups show no differences in the perception of social support between two-parent families and single parents (*χ^2^*(1) = 1.803, *p* = 0.179, *d* = 0.031). However, perceptions of social support differs statistically between two-parent families and large families (*χ^2^*(1) = 4.612, *p* = 0.032, *d* = 0.049), as well as between single parents and large families (*χ^2^*(1) = 6.558, *p* = 0.010, *d* = 0.058). For traditional two-parent families and large families, the results confirm hypothesis H2a2, according to which positive relations exist between social support and the adaptability of families.

Hypothesis H2a1 assumes positive relations between the supportive distribution of roles within the partnership and family adaptability. Analysis of variance revealed no statistically significant differences between the groups in the distribution of the supporting role (*p* = 0.691). In our regression Model 4 (see Table 2), we added the perception of a supportive role distribution within the partnership. Due to the relationship status, we only conducted these analyses for traditional two-parent families and large families. Neither in traditional two-parent families nor in large families was the perceived supportive role distribution within the partnership significantly related to parents’ changes in HLA during the first lockdown. For two-parent families the variance remained the same (4%). For large families, Model 4 explains minimally more variance despite significant correlations (24%). Aligned with the SRMR value the model fit was acceptable (*χ^2^* = 280.06, *df* = 22, RMSEA = 0.15, CFI = 0.00, TLI = 0.00, SRMR = 0.07). The post hoc analysis of the regression coefficients of the perceived supportive role distribution within the partnership between the two family types shows no statistically significant differences (*χ^2^*(1) = 0.084, *p* = 0.772, *d* = 0.007). These results cannot confirm hypothesis H2a1.

## 5. Discussion

In Germany, restrictions to contain the COVID-19 pandemic les to severe disruptions for parents with children under the age of six. These families were denied centralized care and education services, so they needed to reorganize working and childcare hours, and redistribute childcare at home. Our aim was to examine the adaptability of families with young children in times of crisis, with a special focus on adaptability among traditional two-parent families, single parents, and large families. As an indicator of family adaptability, we examined changes in the frequency of parents’ HLA with their children during the first lockdown compared to the preceding time period. Results show, with minor differences, that parents from all three family types offered HLA slightly more often during the first lockdown than before. Since a large proportion of the children spent more time at home due to the closed daycare, this does not, initially, seem surprising. Our results do show that the employment of the surveyed parents hardly changed: Nevertheless, we must note that, whether working from home or not, many families had to organize their employment around childcare and sometimes work early in the morning or late in the evening. To what extent there was enough time for relaxation or family time is questionable. Nevertheless, all parents offered their children HLA more often during the lockdown. It is possible that families tried to compensate for the loss of the influence of early childhood education institutions by providing more HLA. Still, the results revealed significant differences among the groups: Despite fewer personal resources, the single parents in our study, just like families with two parents, managed to offer their children HLA more often compared to before the first lockdown. Adaptability during the first lockdown was comparatively lowest in large families, which identifies this family type as a special risk group in our study.

### 5.1. Adaptability of families in times of crisis: Risk and protective factors

Toward a deeper understanding of family type-specific conditions for adaptability, the identification of factors that influence the adaptability of families is crucial. How parental stress predicts inadequate parenting behavior is well established (Crnic and Greenberg, 1990; Crnic et al., 2005). Overall, studies showed that parental stress is negatively associated with changes in the provision of HLA, in general and especially during the first lockdown in Germany (Barnett, 2008; Oppermann et al., 2021). We were able to confirm this and even show that in large families the tipping point for negative relations between stress and parents’ provision of HLA, as documented by Oppermann et al. (2021), occurs earlier and is stronger overall. Single parents and two-parent families are still relatively adaptable when exposed to the medium stress level. The adaptability of large families, on the other hand, already seems to be vulnerable at a medium stress level. Since these findings just fail the level of significance, follow-up studies are required. Our study initially confirms that high stress restricts the ability to provide HLA in all three family types. There is hardly any variance in our data for child age. We can therefore conclude that the surveyed families were affected by comparable stress when it comes to providing care activities toward their children. We see intermediate negative correlations especially in large families. In the other two family types, the relations are smaller. In large families, the level of parental stress was the best predictor of lower adaptability. Thus, the adaptability of the HLA is significantly more susceptible to stress in large families. This raises the question of whether large families, considered as a special risk group in times of crisis, need special support to compensate for parental stress and thus ensure supportive parenting behavior. In order to make more precise statements about changes in HLA and the associated adaptability of families, it is be necessary to have further information on the general extent of HLA before the lockdown. In general, we were able to show that the relation between stress and the provision of HLA is smallest in two-parent families. It seems that this type of family in particular is the – comparatively – most stress-resistant. This is possibly due to a more balanced distribution of parents and children within this family setting. Compared to the adaptation performance in single-parent families, the availability of parental resources was not a decisive factor for stress management during the lockdown. Of more importance perhaps was how families organized their everyday life, or whether new routines developed during this time. Last but not least, it would also be interesting to analyze the extent to which our results on the stress level of families would be applicable if the target children were older or younger.

As already outlined, social support is crucial to successful adaptability because the extent to which a family feels supported by its social environment contributes to how strongly the emotional burden of a crisis feels (Dunn et al., 2001; Wierda-Boer et al., 2008; Manning et al., 2011). Our study found that the strongest relations between social support and parents’ provision of HLA occur in large families. In line with Oppermann et al. (2021), we thus demonstrate the importance of social support for the adaptability of families and show once again that this predictor seems to be particularly important for an adequate adaptability in large families. If those families receive the level of social support they need for adequate adaptability, this may help to maintain the educational opportunities of children from large families during times of crisis. Moreover, even if the relation between social support and changes in HLA is low in traditional two-parent families, we still see that relations between stress and changes in HLA decrease here. The study by Oppermann et al. (2021) also revealed that social support has a mitigating effect on negative relations between stress and parents’ provision of HLA. Although all three family types perceived similar availability of social support, among single parents, we could not demonstrate relations between social support and changes in HLA. Previous findings considered the lack of social support as the greatest risk factor in single parent adaptability (Jackson and Kiehl, 2017; Martin-West, 2019). It is conceivable that the single parents in our study were already making extensive use of social support before the lockdown and therefore we could find no relations. At the same time, our results show that the employment of single parents scarcely changed and changes in hours of childcare outside the home were been less drastic. Due to their single-parent status, many single parents were entitled to emergency childcare from daycare centers. Problems reconciling work commitments and childcare may have been mitigated in this way, making the importance of social support less relevant during the first lockdown.

As the identification of family resources plays an important role in adaptability (Patterson, 2002), we expected the supportive role distribution within the partnership to be an important predictor of the adaptability of the surveyed families. For changes in HLA during the lockdown, this internal family resource does not seem to have been of importance. The reason for this could be the operationalization of the scale: We surveyed role distribution within the partnership based on self-assessment by the parents. The perception of role distribution alone does not allow any conclusions about the actual distribution of household and care activities. Perhaps it is not the satisfaction with the role distribution that is decisive, but how much parents find fulfilment in their own role.

### 5.2. Further influencing factors

One relation we did not expect concerns gender differences in single-parent families: Single fathers reported a greater increase in HLA than single mothers. The COVID-19 pandemic has certainly led to single mothers and single fathers spending more time with their children. Concerning the time spent on childcare, single fathers often spend less time on childcare than single mothers (Hook and Chalasani, 2008). The single fathers in our study seem to have caught up in terms of time spent with their children during the lockdown. However, when we consider high stress, the gender differences among single parents disappear. Thus, it is a spurious relation between gender and the change in HLA in single-parent families. Rather, the relation is mediated by the level of parental stress. Compared to other family types, there is hardly any well-founded knowledge about the characteristics of single-father families with young children. Mostly, it is older children who live with single fathers. Our findings provide interesting insights into the group of single fathers in early childhood. The fact that single fathers of young children are represented in the sample at all can be seen as a particularly valuable aspect of the study. On the other hand, the results once again make it clear that single mothers in particular are burdened by parental stress. Studying differences between single mothers and fathers is an important area of research to explore child development in different contexts. However, further research on differences between single mothers and fathers needs to examine other aspects of the family environment. The number and age of other children living in the household must also be taken into account in such comparative studies, or whether both family types employ different strategies in obtaining support from their friends and family.

### 5.3. Limitations

The data for the present study come from a Germany-wide cross-sectional survey conducted online, resulting in a convenience sample. Accordingly, parents with a low level of education and single parents are underrepresented, within the sample compared to the German average (Statistisches Bundesamt, 2021). Furthermore, it is not possible to map the extent to which job-seeking participants devote time resources to their job search. Therefore, we cannot assume that those respondents have more time to provide HLA for their children. We also note that some background characteristics of the family situation of the target children (e.g., possible number of younger siblings) cannot be represented. Furthermore, we could not survey changes in HLA directly due to the cross-sectional survey. We asked only for changes in HLA compared to the time before the first lockdown. Consequently, statements can only be made about the changes in HLA as a result of the first COVID-19 lockdown. This also means that no information is available on the initial frequency of HLA. The present findings can therefore be interpreted in terms of how perceived roles in partnership, parental stress, and social support affected changes in HLA, but cannot be generalized to absolute frequencies of HLA. Nor can we make any statements about the quality of the home learning stimulation, such as the atmosphere in a family. The study cannot answer how this changed during the lockdown. The information on changes in HLA is also based on parents’ self-reports, which may be biased by social desirability. Further limitations concern the cross-sectional data as well as the sizes of the regression coefficients. On the one hand, cross-sectional data based on a one-time survey do not allow for causal conclusions. On the other hand, most of the results presented have both low effect sizes and small regression coefficients. This does not make them any less interesting, but we must interpret them with caution. Lastly, we want to point out that the situation of families during the first lockdown was different from the later stages of the COVID-19 pandemic. During the early phase of the COVID-19 pandemic, many families experienced the social shutdown as an opportunity to unwind, and optimism about overcoming the crisis was certainly higher than after many months of lockdown. Families were able to use this time to develop a more mindful family life, establish new rituals, and strengthen their own relationships (Weissbourd et al., 2020). This raises the question of whether our findings are also tenable over a longer period.

## 6. Conclusions and implications

This article contributes to linking the dynamics of HLE more closely to adaptation processes in families. We were thereby able to explain relations between perceived partnership roles, parental stress or social support and the adaptability of families in times of crisis. The present results illustrate that different resources of different types of family were indeed relevant for their adaptability during the COVID-19 lockdown in Germany. This becomes particularly apparent when it comes to the adaptability of families in times of crisis. We were able to show that the adaptation of HLA during the lockdown differed slightly based on family type, and that depending on family type distinct predictors are important for successful adaptability. For two-parent families and single parents, our analyses explained little variance. How these families can be better supported in their adaptability, and which predictors are important here, must be investigated in further studies in depth. Furthermore, follow-up studies should consider statements about the family appraisal component. This would allow links between the importance of parental self-efficacy for HLE and adaptability in times of crisis. Still, particularly noteworthy here are the results for large families. The study makes clear that special attention should be paid to these families in times of crisis and raises the question of whether the special needs of large families have received enough attention within social and political debates during the first lockdown in Germany. In times of crisis, it is important to ensure that all families receive the support they need to care for their children in the best possible way. To do this, we need further research to give a clear picture of where requirements remain unfulfilled, and what the special needs of individual families are. The characteristics of specific types of family should not be neglected in these considerations; they can further our understanding of the dynamics in different family types, which is essential for the development of effective and needs-oriented family support programs and policies.

## Data availability statement

The datasets presented in this article are not readily available because the data are currently reserved for scientific qualifications (Ph.D. and masters’ theses). Requests to access the datasets should be directed to EO, elisa.oppermann@uni-bamberg.de.

## Author contributions

LP: conceptualization, methodology, formal analysis, writing–original draft, and visualization. FC: conceptualization, methodology, data collection, and writing–review and edit. EO: conceptualization, methodology, data collection, writing–review and editing. YA: conceptualization, methodology, writing–review and editing, supervision, and resources. All authors contributed to the article and approved the submitted version.

## Conflict of interest

The authors declare that the research was conducted in the absence of any commercial or financial relationships that could be construed as a potential conflict of interest.

## Publisher’s note

All claims expressed in this article are solely those of the authors and do not necessarily represent those of their affiliated organizations, or those of the publisher, the editors and the reviewers. Any product that may be evaluated in this article, or claim that may be made by its manufacturer, is not guaranteed or endorsed by the publisher.

## Figures and Tables

**Table:** 

Pearson correlations								
	Age of child	Perceived financial problems before COVID-19	ISCED	Change in employment (household level)	Change in hours of childcare outside the home	Parental stress	Social support	Supportive role distribution partnership
Perceived financial problems before COVID-19	−0.104^**^							
ISCED	0.156^**^	−0.172^**^						
Change in employment (household level)	0.032^**^	−0.045^**^	0.050^**^					
Change in hours of childcare outside the home	−0.056^**^	0.054^**^	−0.150^**^	0.087^**^				
Parental Stress	0.029^*^	0.084^**^	0.035^**^	0.040^**^	−0.095^**^			
Social support	−0.065^**^	−0.084^**^	0.008	0.005	0.079^**^	−0.374^**^		
Supportive role distribution partnership	0.007	−0.119^**^	0.052^**^	0.019	−0.018	−0.161^**^	0.199^**^	
Changes in parents’ HLA with their children	−0.048^**^	−0.060^**^	0.038^**^	−0.046^**^	−0.082^**^	−0.134^**^	0.105^**^	0.056^**^

**The correlation is significant at the 0.01 level (2-sided).

*The correlation is significant at the 0.05 level (2-sided).
